# Editorial: Unravelling the basis of non-invasive prenatal screening results

**DOI:** 10.3389/fgene.2023.1247764

**Published:** 2023-07-17

**Authors:** Luigia De Falco, Elisabetta Pelo, Zhongxia Qi, Antonio Novelli

**Affiliations:** ^1^ AMES, Centro Polidiagnostico Strumentale, srl, Naples, Italy; ^2^ SOD Diagnostica Genetica, Azienda Ospedaliero Universitaria Careggi, Florence, Italy; ^3^ Medical Center, Department of Laboratory Medicine, University of California, San Francisco, San Francisco, CA, United States; ^4^ Translational Cytogenomics Research Unit, Bambino Gesù Children’s Hospital, IRCCS, Rome, Italy

**Keywords:** prenatal diagnosis, non-invasive prenatal screening, discordant results, fetoplacental chromosomal mosaicism, twin pregnancies

The presence of circulating cell-free DNA (cfDNA) from the placenta in the maternal circulation was first demonstrated by Lo et al. (Lo et al., 1997). Since its commercial launch in 2011, cfDNA-based non-invasive prenatal testing (NIPT) has permitted screening for T21, T18, and T13 with high specificity and sensitivity in both high-and low-risk populations (La Verde et al., 2021).

Circulating cell-free DNA in pregnant women is a mixture of maternal and placental cell-free DNA, in which the maternal fraction is on average ten times the fetal one (fetal fraction, FF). Hence, false-positive, false-negative as well as non-reportable cases exist and may due to technical issues or may be attributable to biological causes such as low fetal fraction, feto-placental mosaicism, or vanishing twin (Grati, 2014; 2016; Bianchi and Chiu, 2018; Deng and Liu, 2022). This Research Topic Unravelling the basis of non-invasive prenatal screening results collect some recent papers focused on discordances between non-invasive prenatal screening result and fetal karyotype with emphasis on chromosomal mosaicisms. Chromosomal mosaicism (CM) is a biological phenomenon in human and is found in approximately 1%–4% of prenatal diagnosis performed by chorionic villus sampling and in about 0.1%–0.3% of amniocentesis (Hsu et al., 1996; Grati et al., 2017; Lund et al., 2020). As reported by Li et al., CM is still one of the main difficult Research Topic in prenatal diagnosis due to the uncertainty outcome, especially when fetal ultrasonographic features appear normal and the use of multiple methods, such as a combination of karyotyping, and fluorescent *in situ* hybridization (FISH) was recommended. Moreover, CMA combined with karyotyping can be recommended as the preferred method of prenatal diagnosis for cases where NIPS results indicate a high risk in pregnancy as suggested by Bu et al. In this context the classic karyotype analysis and NIPT analysis are limited in determining the mosaic sex chromosomal abnormalities (Ma et al., 2021). On the contrary, single nucleotide polymorphism (SNP) array is validated in detecting the chromosomal syndromes, mosaic chromosomal syndromes as well as chromosomal deletions/duplications with high accuracy and high resolution (Samango-Sprouse et al., 2013). Wang et al. reported a retrospective investigation of sex chromosomes anomalies in Fujian Province cohort by SNP array, showing the importance of using different technologies to define segmental aneuploidies. False negative NIPT results, that have the highest clinical impact on patients and clinicians, are mainly due to placental mosaicisms. Feresin et al., reported two cases of feto-placental mosaicism of trisomy 21, both with a low-risk NIPT result, identified by ultrasound signs and a subsequent amniocentesis consistent with a trisomy 21. In both cases, cytogenetic and/or cytogenomic analyses were performed on the placenta and fetal tissues, showing in the first case a mosaicism of trisomy 21 in both the placenta and the fetus, but a mosaicism in the placenta and a complete trisomy 21 in the second case. In addition, Bonanni et al., reported a case of CPM in which a NIPT false-positive result for trisomy 13 required two further invasive diagnostic tests–an amniocentesis and a cordocentesis–to rule out the fetal aneuploidy. In this paper the authors showed that given the trophoblastic origin of cf-DNA, NIPT is a screening test and the real benefit of cfDNA analysis lies, therefore, in its complementary use with ultrasound scan, Therefore, NIPT remains a powerful tool allowing non-invasive access to the cytotrophoblast. In this regard, Kleinfinger et al. showed that genome-wide NIPT can be used to characterize the supernumerary marker chromosomes (SMCs) revealed by karyotyping of chorionic villi, effectively guiding the choice of further genomic analyses and reducing the period of uncertainty for the patient. They were able to carry out targeted FISH resulting in rapid, effective, and accurate characterization of the SMCs and their distribution in the fetoplacental unit, ultimately allowing determination of their clinical significance. In contrast to chorionic villus sampling (CVS), an invasive diagnostic technique that samples a small region of the placenta, NIPT noninvasively assesses the genetic status of the cytotrophoblast as a whole. These cases emphasize the need for accurate and complete pre-test NIPT counselling, as well as for molecular studies of placenta and fetal tissue in order to discriminate between placental, fetal and feto-placental mosaicism, and between complete or mosaic fetal chromosomal anomalies.

As the cfDNA in the maternal plasma fraction originates from the cytotrophoblast of chorionic villi (CV), a high-risk call for a rare autosomal aneuploidy (RAA) may be indicative of confined placental mosaicism (CPM) and not true fetal aneuploidy. In more recent years, the use of cfDNA screening has been expanded to genome-wide screening for RAAs and partial deletions and duplications (i.e., copy number variants, including selected microdeletions) and an increasing number of studies have described the test performance and the clinical validity of these applications (Pescia et al., 2017; Pertile et al., 2021; Soster et al., 2021; van Prooyen Schuurman et al., 2022). The screen-positive rate for RAAs has been shown to range from 0.12% (Scott et al., 2018) to 1.1% (Van Opstal et al., 2020). In this Research Topic Mossfield et al. described a cohort of pregnancies with a NIPT high risk result for the presence of a RAA. Follow up information was available in 68% (74/109) of the patients with a concordance rate of 20.3%, i.e., the presence of a RAA was confirmed in 15/74. Intrauterine fetal demise, fetal growth restriction, and preterm birth, were observed both in patients with fetal or placental confirmation of the presence of a RAA, as well as patients that did not undergo fetal and/or placental diagnostic testing. Furthermore, the Authors proposed that genome-wide cfDNA screening for RAA can in some cases provide useful information for pregnancy management and counselling giving a possible explanation for adverse pregnancy outcome.

Although the recent ACMG guidelines note that at this time there is insufficient evidence to either recommend or not recommend NIPT for the identification of RAA and CNV (Dungan et al., 2023), and the ISPD position statement not recommend NIPT for the identification of RAA and CNV for the routine care of unselected populations (Hui et al., 2023), some studies explored the attitudes and preferences of patients regarding expanded NIPT. In this Research Topic Dubois et al. examined the attitudes and preferences on expanded NIPT of pregnant women having first-tier cfDNA screening at a private prenatal clinic in Canada, including the main factors influencing the decision-making process undergoing expanded cfDNA screening. Their findings suggest that with appropriate pre-test counseling, pregnant women may choose NIPT for an expanding list of conditions, even if, they should be made aware of both the benefits and limitations of expanded NIPT and the possibility of discordant/inconclusive results.

Therefore, development of reliable synthetic materials available for NIPS is necessary for validation steps and quality assessment in laboratories providing this test. Although synthetic positive plasmas are commercially available, they are usually insufficient for the initial validation due to limited abnormality types and sample quantity. In the paper Qi et al., described a simple method of making synthetic positive plasmas that are reliable and excellent alternatives of positive maternal plasmas for validation and monitoring NIPS performance.

Another interesting topic is the application of NIPT in multiple pregnancies. The rates of twin pregnancies have increased over the last four decades in many countries, likely due to several factors including increased maternal age at birth and the increased use of assisted reproductive techniques (Pison et al., 2015; Palomaki et al., 2021). Multifetal pregnancies are at increased risk for a broad range of pregnancy complications and adverse outcomes, and the primary associated risk factor for a poor pregnancy outcome in twin pregnancies is the chorionicity. Zygosity can be established using NIPT and this can be particularly useful when there are concerns about chorionicity or determining whether one *versus* two fetuses are affected (Norwitz et al., 2019; Benn and Rebarber, 2021). Guo et al., presented a rare case in which an IVF-ET twin pregnancy gave birth to a partial trisomy 21 chimera girl in which both Nuchal translucency (NT) and NIPT had limitations in detecting the trisomy 21 mosaicism in a twin pregnancy. Hence, the results from this case report indicate that IVF-ET pregnancies should be strictly monitored by ultrasound and obstetric follow up also to exclude false negative results.

## References

[B1] Benn P. Rebarber A. (2021). Non‐invasive prenatal testing in the management of twin pregnancies. Prenat. Diagn. 41, 1233–1240. 10.1002/pd.5989 34170028 PMC8518532

[B2] Bianchi D. W. Chiu R. W. K. (2018). Sequencing of circulating cell-free DNA during pregnancy. N. Engl. J. Med. 379, 464–473. 10.1056/NEJMra1705345 30067923 PMC10123508

[B3] Deng C. Liu S. (2022). Factors affecting the fetal fraction in noninvasive prenatal screening: A review. Front. Pediatr. 10, 812781. 10.3389/fped.2022.812781 35155308 PMC8829468

[B4] Dungan J. S. Klugman S. Darilek S. Malinowski J. Akkari Y. M. N. Monaghan K. G. (2023). Noninvasive prenatal screening (NIPS) for fetal chromosome abnormalities in a general-risk population: An evidence-based clinical guideline of the American College of Medical Genetics and Genomics (ACMG). Genet. Med. 25, 100336. 10.1016/j.gim.2022.11.004 36524989

[B5] Grati F. R. (2014). Chromosomal mosaicism in human feto-placental development: Implications for prenatal diagnosis. J. Clin. Med. 3, 809–837. 10.3390/jcm3030809 26237479 PMC4449651

[B6] Grati F. R. (2016). Implications of fetoplacental mosaicism on cell-free DNA testing: A review of a common biological phenomenon. Ultrasound Obstet. Gynecol. 48, 415–423. 10.1002/uog.15975 27240559

[B7] Grati F. R. Malvestiti F. Branca L. Agrati C. Maggi F. Simoni G. (2017). Chromosomal mosaicism in the fetoplacental unit. Best Pract. Res. Clin. Obstetrics Gynaecol. 42, 39–52. 10.1016/j.bpobgyn.2017.02.004 28284509

[B8] Hsu L. Y. F. Yu M.-T. Richkind K. E. Van Dyke D. L. Crandall B. F. Saxe D. F. (1996). Incidence and SIGNIFICANCE OF chromosome mosaicism involving an autosomal structural abnormality diagnosed prenatally through amniocentesis: A collaborative study. Prenat. Diagn. 16, 1–28. 10.1002/(SICI)1097-0223(199601)16:1<1::AID-PD816>3.0.CO;2-W 8821848

[B9] Hui L. Ellis K. Mayen D. Pertile M. D. Reimers R. Sun L. (2023). Position statement from the International Society for Prenatal Diagnosis on the use of non‐invasive prenatal testing for the detection of fetal chromosomal conditions in singleton pregnancies. Prenat. Diagn. 43, 814–828. 10.1002/pd.6357 37076973

[B10] La Verde M. De Falco L. Torella A. Savarese G. Savarese P. Ruggiero R. (2021). Performance of cell-free DNA sequencing-based non-invasive prenatal testing: Experience on 36,456 singleton and multiple pregnancies. BMC Med. Genomics 14, 93. 10.1186/s12920-021-00941-y 33785045 PMC8011149

[B11] Lo Y. M. Corbetta N. Chamberlain P. F. Rai V. Sargent I. L. Redman C. W. (1997). Presence of fetal DNA in maternal plasma and serum. Lancet 350, 485–487. 10.1016/S0140-6736(97)02174-0 9274585

[B12] Lund I. C. B. Becher N. Christensen R. Petersen O. B. Steffensen E. H. Vestergaard E. M. (2020). Prevalence of mosaicism in uncultured chorionic villus samples after chromosomal microarray and clinical outcome in pregnancies affected by confined placental mosaicism. Prenat. Diagn. 40, 244–259. 10.1002/pd.5584 31769052

[B13] Ma N. Xi H. Chen J. Peng Y. Jia Z. Yang S. (2021). Integrated CNV-seq, karyotyping and SNP-array analyses for effective prenatal diagnosis of chromosomal mosaicism. BMC Med. Genomics 14, 56. 10.1186/s12920-021-00899-x 33632221 PMC7905897

[B14] Mossfield T. Soster E. Menezes M. Agenbag G. Dubois M.-L. Gekas J. (2022). Multisite assessment of the impact of cell-free DNA-based screening for rare autosomal aneuploidies on pregnancy management and outcomes. Front. Genet. 13, 975987. 10.3389/fgene.2022.975987 36105088 PMC9465083

[B15] Norwitz E. R. McNeill G. Kalyan A. Rivers E. Ahmed E. Meng L. (2019). Validation of a single-nucleotide polymorphism-based non-invasive prenatal test in twin gestations: Determination of zygosity, individual fetal sex, and fetal aneuploidy. JCM 8, 937. 10.3390/jcm8070937 31261782 PMC6679081

[B16] Palomaki G. E. Chiu R. W. K. Pertile M. D. Sistermans E. A. Yaron Y. Vermeesch J. R. (2021). International society for prenatal diagnosis position statement: Cell free (cf) DNA screening for down syndrome in multiple pregnancies. Prenat. Diagn. 41, 1222–1232. 10.1002/pd.5832 33016373

[B17] Pertile M. D. Flowers N. Vavrek D. Andrews D. Kalista T. Craig A. (2021). Performance of a paired-end sequencing-based noninvasive prenatal screening test in the detection of genome-wide fetal chromosomal anomalies. Clin. Chem. 67, 1210–1219. 10.1093/clinchem/hvab067 34077512

[B18] Pescia G. Guex N. Iseli C. Brennan L. Osteras M. Xenarios I. (2017). Cell-free DNA testing of an extended range of chromosomal anomalies: Clinical experience with 6,388 consecutive cases. Genet. Med. 19, 169–175. 10.1038/gim.2016.72 27362910 PMC5303761

[B19] Pison G. Monden C. Smits J. (2015). Twinning rates in developed countries: Trends and explanations. Popul. Dev. Rev. 41, 629–649. 10.1111/j.1728-4457.2015.00088.x

[B20] Samango-Sprouse C. Banjevic M. Ryan A. Sigurjonsson S. Zimmermann B. Hill M. (2013). SNP-based non-invasive prenatal testing detects sex chromosome aneuploidies with high accuracy: Non-invasive prenatal testing detects sex chromosome aneuploidies. Prenat. Diagn 33, 643–649. 10.1002/pd.4159 23712453 PMC3764608

[B23] Scott F. Bonifacio M. Sandow R. Ellis K. Smet M. E. McLennan A. (2018). Rare autosomal trisomies: Important and not so rare. Prenat. Diagn. 38 (10), 765–771. 10.1002/pd.5325 29956348

[B21] Soster E. Boomer T. Hicks S. Caldwell S. Dyr B. Chibuk J. (2021). Three years of clinical experience with a genome-wide cfDNA screening test for aneuploidies and copy-number variants. Genet. Med. 23, 1349–1355. 10.1038/s41436-021-01135-8 33731879 PMC8257487

[B24] Van Opstal D. Eggenhuizen G. M. Joosten M. Diderich K. Govaerts L. Galjaard R. J. (2020). Noninvasive prenatal testing as compared to chorionic villus sampling is more sensitive for the detection of confined placental mosaicism involving the cytotrophoblast. Prenat. Diagn. 40 (10), 1338–1342. 10.1002/pd.5766 32533714 PMC7540368

[B22] van Prooyen Schuurman L. Sistermans E. A. Van Opstal D. Henneman L. Bekker M. N. Bax C. J. (2022). Clinical impact of additional findings detected by genome-wide non-invasive prenatal testing: Follow-up results of the TRIDENT-2 study. Am. J. Hum. Genet. 109, 1140–1152. 10.1016/j.ajhg.2022.04.018 35659929 PMC9247828

